# Elevated Pentraxin 3 in bone metastatic breast cancer is correlated with osteolytic function

**DOI:** 10.18632/oncotarget.1664

**Published:** 2014-01-15

**Authors:** Bongkun Choi, Eun-Jin Lee, Da-Hyun Song, Sung-Chul Yoon, Yeon-Ho Chung, Youngsaeng Jang, Sang-Min Kim, Youngsup Song, Sang-Wook Kang, Seung-Yong Yoon, Eun-Ju Chang

**Affiliations:** ^1^ Department of Biomedical Sciences, University of Ulsan College of Medicine, Asan Medical Center, Seoul, Korea; ^2^ Division of Biostatistics, Center for Medical Research and Information, University of Ulsan College of Medicine, Asan Medical Center, Seoul, Korea; ^3^ Department of Anatomy and Cell Biology, University of Ulsan College of Medicine, Asan Medical Center, Seoul, Korea; ^4^ Cellular Dysfunction Research Center and BMIT, University of Ulsan College of Medicine, Seoul, Korea

**Keywords:** breast cancer, PTX3, osteoclast, RANKL, osteolysis

## Abstract

Pentraxin 3 (PTX3), a modulator of tumor-associated inflammation, is known to be positively correlated with tumor grade and severity of malignancies, but its exact role remains unclear. This study found that PTX3 expression was up-regulated in distant bone metastases of breast cancer compared to lung, liver, and brain metastases in 64 human breast cancer patients. Elevated expression of PTX3 was correlated with poor survival in patients with breast cancer. PTX3 expression was also up-regulated in a bone metastatic breast cancer cell line and further enhanced by pro-inflammatory cytokine TNFα. Administration of PTX3 promoted the migratory potential of breast cancer cells and the mobilization of macrophages, a precursor of osteoclasts (OCs), toward breast cancer cells. In addition, elevated expression of PTX3 by TNFα led to enhanced OC formation, implying the distinct role of PTX3 in osteolytic bone metastasis of breast cancer cells. Furthermore, PTX3 silencing using siRNA-specific siRNA prevented breast cancer cell migration, macrophage Chemotaxis, and subsequent OC formation. These findings provide an important insight into the key role of PTX3 in inflammation-associated osteolytic complications of breast cancer.

## INTRODUCTION

Bone is a unique environment storing a variety of growth factors, and is one of the most common target sites for distant metastasis of breast cancer [1]. Approximately 80% of patients with disseminated metastatic breast cancer develop skeletal metastases [2, 3], emphasizing that breast cancer has a propensity to metastasize to bone. Bone metastases from breast cancer are typically osteolytic and feature bone resorption [4–8]. In the bone microenvironment, breast cancer cells produce osteoclast (OC)-activating cytokines, including parathyroid hormone-related protein (PTH-rP), prostaglandin E_2_ (PEG_2_), and interleukin-11 (IL–11) [5], which can increase expression of receptor activator of nuclear factor-κB ligand (RANKL) by osteoblasts (OBs). RANKL is a potent stimulator of OC differentiation and OC activity, and leads to excess bone destruction [5, 6]. As a result, OCs degrade bone and release a variety of bone-storing growth factors, such as insulin-like growth factors (IGF), transforming growth factor-beta (TGF-beta), fibroblast growth factor (FGF), platelet-derived growth factor, and bone morphogenic proteins (BMPs) from bone [5–8]. These growth factors facilitate tumor growth, thus establishing a “vicious cycle” [7, 8]. Metastases to bone worsen quality of life in these patients by causing pathological manifestations of osteolytic lesions, including devastating bone pain, pathological fractures, spinal compression, and hypercalcemia, which indirectly lead to earlier death [9]. Therefore, early detection of bone metastases and an increased understanding of the cellular and molecular mechanisms contributing to osteolysis will improve patients' quality of life and decrease morbidity and mortality [10].

Breast cancer cells that have metastasized to bone act as the main drivers of the pro-inflammatory, cytokine-rich environment of the bone, associated with bone destruction in breast cancer patients [7, 11–14]. Inflammatory cells and mediators (e.g., cytokines and chemokines) are present in the tumor microenvironment and have crucial roles at different stages of tumor development [15, 16]. Inflammatory cytokines, including tumor necrosis factor alpha (TNFα), interleukin-1beta (IL-1β) and IL-6, increase the invasive capacity of cancer cells [15]. Necrotic cell death and hypoxia trigger the release of inflammatory mediators and recruitment of leukocyte, such as macrophages, precursors of OCs [17–20]. During metastasis, pro-inflammatory cytokines and chemokines enhance interactions between cancer cells and infiltrating leukocytes and these interactions in turn promote the migration, invasion, and survival of cancer cells in distant organs, facilitating colonization of tumor cells at the metastatic niche [21, 22]. Thus, identifying the inflammatory mediators produced by metastatic cancer cells, and which drive the vicious cycle of osteolysis in the context of macrophage migration, tumor growth, and OC formation, is of considerable interest.

PTX3, a modulator of inflammatory processes, is a member of the highly conserved pentraxin superfamily, which includes C-reactive protein (CRP) [23, 24]. PTX3 expression is induced in various cell types under the control of several mediators, including pro-inflammatory cytokines (e.g., IL-lβ, TNFα), tissue damage, and microbes [25, 26]. PTX3 contributes to the regulation of inflammation and complement activation, and also participates in tissue remodeling and modification of angiogenesis [27–31]. PTX3 has been suggested to play a significant role in tumor-associated inflammation and was shown to be up-regulated in several malignancies, including liposarcoma [32], lung cancer [33], pancreatic cancer [34], and glioma [35]. In a recent study, Kondo et al. reported that extracellular PTX3 protein promotes migratory potential of pancreatic cancer cells, and that PTX3 expression significantly associates with advanced clinical stage of pancreatic cancer patients [34]. PTX3 expression also positively correlates with tumor grade and severity in glioma, representing PTX3 as a marker of cancer-related inflammation and malignancy [35]. Furthermore, it has been suggested that PTX3 is an informative serum biomarker of lung carcinoma, being highly expressed in high risk lung cancer patients [33] and over-expressed in human soft tissue liposarcoma [36] and prostate tumor [37]. Thus, PTX3 is a potent candidate marker of inflammation [38, 39], including cancer-related inflammation [32, 33, 36, 37]: however, the precise role of PTX3 in cancer progression has not been elucidated.

The present study was undertaken to assess the presence and significance of PTX3 in human breast cancers in the context of bone metastasis, and its related osteolytic function. We addressed the elevated expression and secretion of PTX3 in breast cancer cells with bone metastatic properties compared to non-bone metastases of breast cancer, and its related regulation by TNFα. In addition, we investigated the potent role of PTX3 in promoting the migratory capacity of breast cancer cells, and Chemotaxis of macrophages and precursors of OCs toward breast cancer cells. Finally, we defined the molecular mechanism of PTX3 osteolytic function as increasing osteoblast RANKL production. Our findings provide evidence for the key role of PTX3 and its related mechanism in bone metastasis of breast cancer, possibly reflecting the osteolytic process of breast cancer associated with inflammation.

## RESULTS

### Up-regulation of PTX in bone metastasized tumor tissue in human breast cancer patients and bone metastatic breast cancer cells

**Figure 1:**
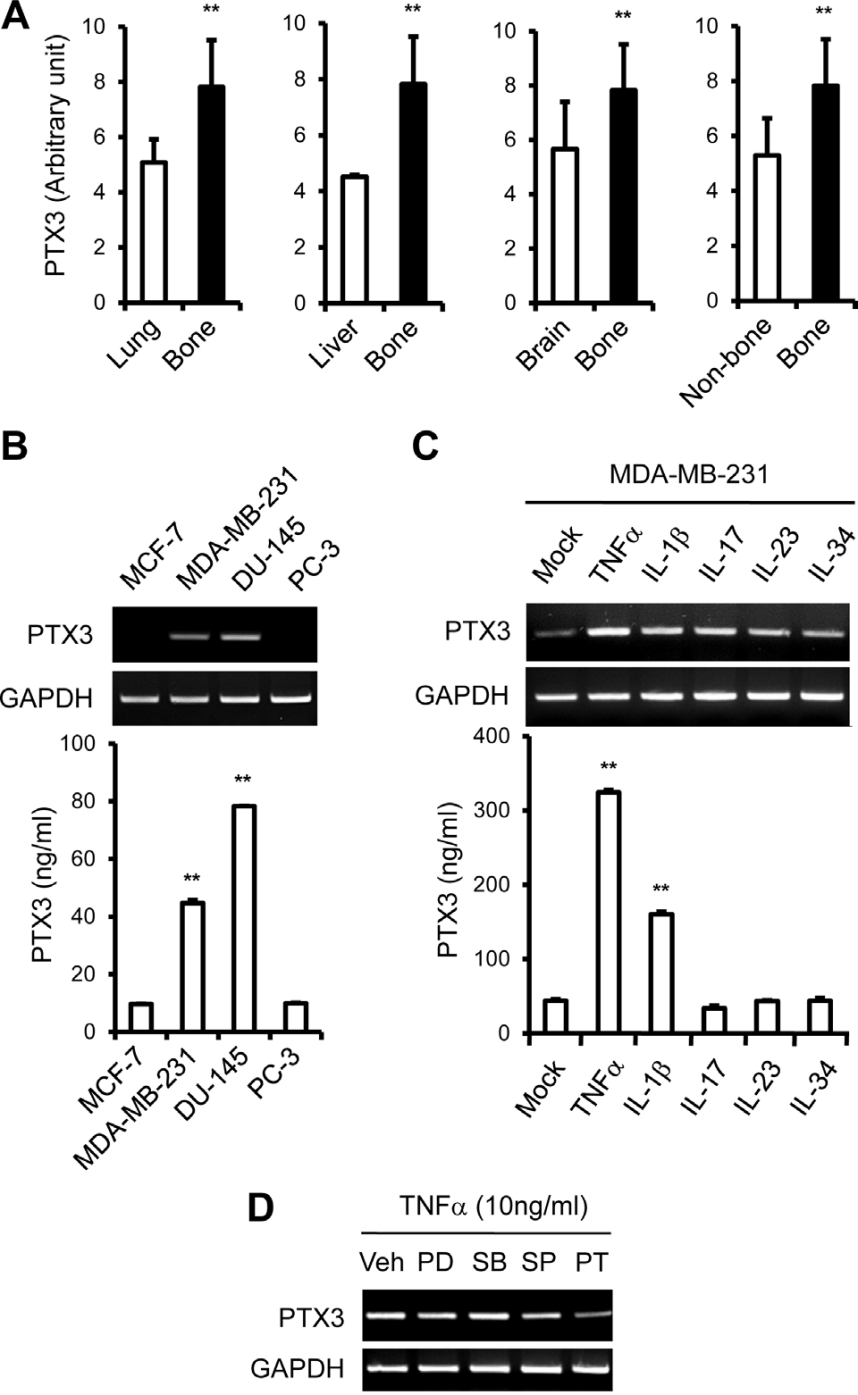
Up-regulation of PTX3 expression in bone metastasized tumor tissue in human breast cancer patients and bone metastatic human breast cancer cells A: Gene expression analysis of PTX3 in distant metastatic tumor tissues in human breast cancer patients (GSE14020). Values for PTX3 mRNA expression were analyzed in lung (n=20), liver (n=5), brain (n=22), or bone (n= 17) metastasized tumor tissues in breast cancer patients. Wilcoxon rank sum tests were performed to compare PTX3 expression in human breast cancer patients. **B:** Cells were lysed and total RNA was extracted as described in the Materials and Methods. PTX3 mRNA levels in human breast (MCF-7 and MDA-MB-231) and prostate cancer (DU-145 and PC-3) cells were determined by reverse transcriptase PCR (RT-PCR). Glyceraldehyde 3-phosphate dehydrogenase (GAPDH) mRNA levels were detected as a control. Culture media were collected and the concentrations of PTX3 protein were measured using an enzyme-linked immunosorbent assay (ELISA) assay. **C:** MDA-MB-231 cells were treated with different cytokines (10 ng/ml of TNF-α, IL-lβ, IL-17, IL-23, and IL-34) for 24 hours and PTX3 mRNA and protein expressions were determined as described in panel B. Bars indicate the mean and standard deviation (SD) of triplicate samples. **D:** Nuclear factor kappa B (NF-κB) dependent PTX3 mRNA expression upon TNFα stimulation in MDA-MB-231 cells. MDA-MB-231 cells were pretreated with the vehicle (dimethyl sulfoxide, 10 μM), the extracellular signal-regulated protein kinase (ERK) inhibitor, PD98059 (10 μM), p38 inhibitor, SB203580 (10 μM), JNK inhibitor, SP600125 (10 μM) or NF-κB inhibitor, pyrrolidine dithiocarbamate (10 μM) for 30 minutes and then treated with 10 ng/ml TNFα for 24 hours. PTX3 mRNA levels were determined in cell lysates by RT-PCR. Veh, vehicle; PD, PD98059; SB, SB203580; SP, SP600125; PT, PDTC. (**P < 0.005, compared to control or none treated).

To investigate the association of the PTX3 gene expression signature with bone metastasis, we analyzed a cohort of 64 breast cancer patients for whom genome-wide gene expression data was available. Analysis of PTX3 expression between bone metastatic and non-bone metastatic relapsed malignant tissues from breast cancer patients was performed using published data from the Gene Expression Omnibus (GEO) [40]. The expression level of PTX3 in bone metastasis sample was significantly higher than that of lung, liver, or brain metastases in breast cancer patients. These results suggested that PTX3 mRNA was over-expressed (*p*<0. 05) in clinical breast tissues with distant bone metastasis compared to those with lung, liver, or brain metastases (Figure 1A). Gene expression data from primary breast cancer patients also displayed associations between PTX3 signature and survival time in a cohort of 203 primary breast cancer tissues of human breast cancer patients (Supplementary Figure S1). Results from a Kaplan-Meier survival analysis showed that high expression of PTX3 associates with poor survival *(*p*=0.052)* (Supplementary Figure S1).

Elevated expression of PTX3 has also been associated with increased risk of liposarcoma, glioma, lung cancer, prostate carcinoma, and pancreatic carcinoma [32–35]. Although PTX3 is expressed in a variety of cells and induced by inflammatory conditions, the role of PTX3 in breast cancer malignancy and metastasis is unclear. Based on the results in Figure 1A, we postulated that bone metastatic breast cancer cells may express higher levels of PTX3 than non-bone metastatic breast cancer cells. PTX3 mRNA expression was significantly increased in the bone metastatic breast cancer cell line MDA-MB-231 compared to the non-bone metastatic breast cancer cell line MCF-7, as shown by RT-PCR (Figure 1B). PTX3 proteins are known to be secreted from cells [41], and the expression levels of PTX3 protein in conditioned media from MCF-7 and MDA-MB-231 cells were measured by enzyme-linked immunosorbent assay (ELISA). The expression level of PTX3 protein was also significantly elevated in MDA-MB-231 compared to MCF-7 cells (*p*<0.005) (Figure 1B). To study tissue specificity, DU-145 and PC-3 prostate cancer cell lines were also analyzed. PC-3 cells are metastatic to bone whereas DU-145 cells are less frequently metastatic to bone. We found that high levels of PTX3 mRNA and protein were expressed in the non-bone metastatic prostate cancer cell line DU-145 compared to bone metastatic prostate cancer cell line PC-3 (Figure 1B), in contrast to breast cancer cells. This indicates that PTX3 is expressed in bone metastatic human breast cancer cells, which are predominantly osteolytic. These results suggest that PTX3 may be a mediator involved in breast cancer mediated osteolysis.

### TNFα up-regulates PTX3 expression in bone metastatic breast cancer cells

Based on these results, and because MDA-MB-231 is a bone metastatic and osteolytic breast cancer cell line, MDA-MB-231 cells were selected for further analysis. Because PTX3 expression is induced in various cell types by several stimuli, including pro-inflammatory cytokines (e.g., IL-lβ, TNFα) [26], we treated MDA-MB-231 cells with a variety of cytokines to determine whether MDA-MB-231 cells produce PTX3 in response to pro-inflammatory cytokines. MDA-MB-231 cells were treated with TNFα, IL-lβ, IL-17, IL-23, and IL-34 (each at 10 mg/ml) for 24 hours. The increase in PTX3 mRNA expression was more marked in TNFα-stimulated cells than IL-17, IL-23, and IL-34-stimulated cells (Figure 1C). IL-lβ also modestly induced PTX3 expression in MDA-MB-231 cells (Figure 1C). The level of secreted PTX3 proteins from cells was also increased approximately sixfold in MDA-MB-231 cells in response to TNFα (Figure 1C). Together, these results suggest that TNFα regulates PTX3 expression in bone metastatic breast cancer cells at both mRNA and protein levels.

TNFα activates nuclear factor-kappa-B (NF-κB) and c-Jun N-terminal kinase (JNK), which are important for TNFα-mediated gene expression and activation of cells [42]. To understand better the mechanism by which TNFα increases PTX3 production in breast cancer cells, we examined the effects of inhibitors of extracellular signal-regulated protein kinase (ERK), p38 mitogen-activated protein kinase (MAPK), JNK, or NF-κB on PTX3 expression. MDA-MB-231 cells were preheated with the vehicle, PD98059 (ERK inhibitor), SB203580 (p38 MAPK inhibitor), SP600125 (JNK inhibitor), or pyrrolidine dithiocarbamate (PDTC; NF-κB inhibitor) at a concentration of 10 μM for 30 minutes, followed by incubation with TNF-α for 24 hours. As shown in Figure 1D, the NF-κB inhibitor PDTC effectively reduced TNF-α-induced PTX3 mRNA expression. However, p38, ERK, or JNK inhibition did not affect TNF-α-induced PTX3 mRNA expression (Figure 1D). Collectively, these results suggest that the TNF-α-induced increase in PTX3 expression in MDA-MB-231 is mediated by NF-κB pathways.

**Figure 2:**
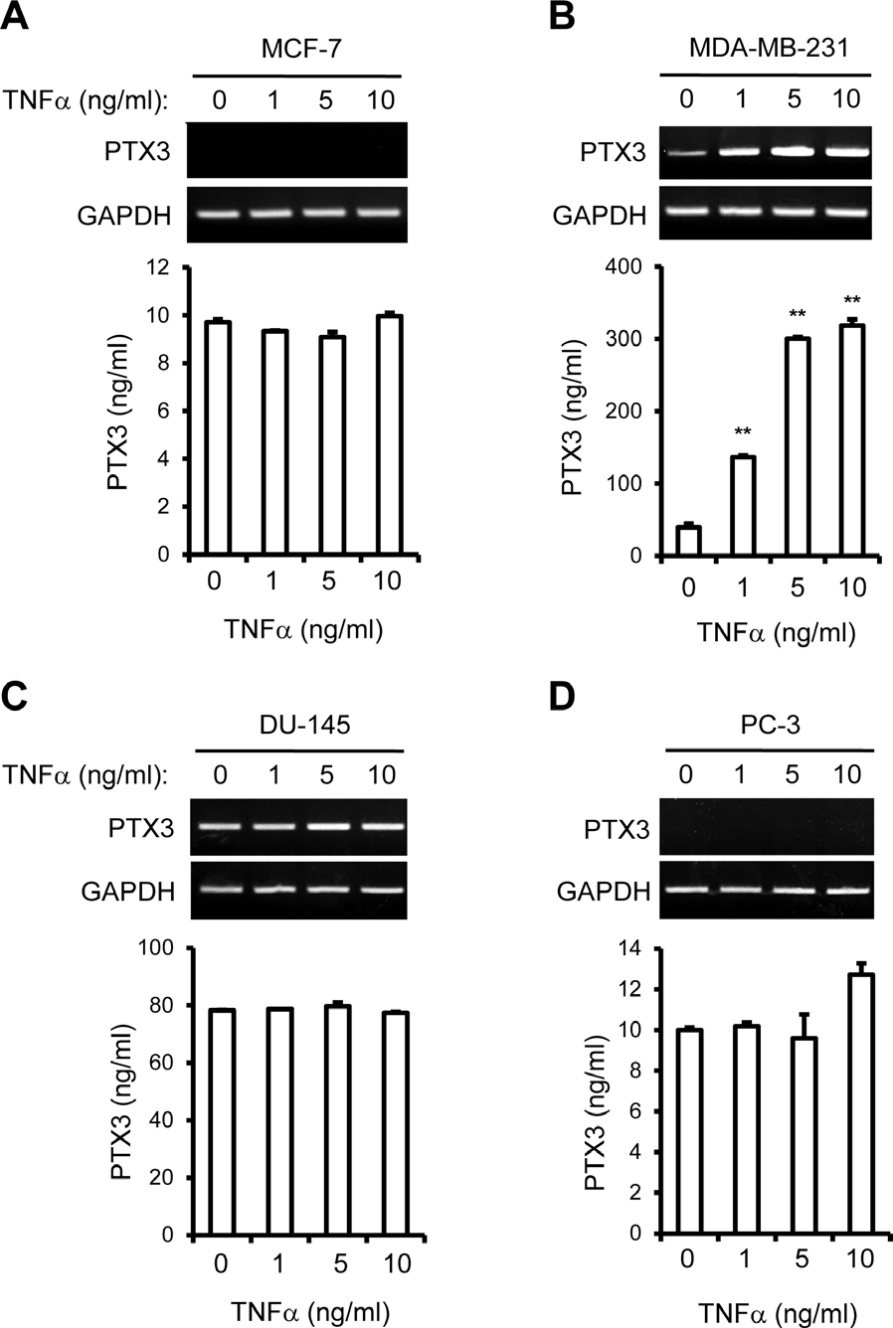
Tumor necrosis factor alpha (TNFα) up-regulates the expression of PTX3 in bone metastatic breast cancer cells MCF-7 (A) and MDA-MB-231 (B) of breast cancer cells and DU-145 (C) and PC-3 (D) of prostate cancer cells were cultured with various concentrations of TNFα for 24 hours. PTX3 mRNA mRNA levels in cell lysates were determined by RT-PCR and the concentrations of PTX3 protein in the media were measured by ELISA as described in Figure 1. Glyceraldehyde 3-phosphate dehydrogenase (GAPDH) mRNA levels were detected as a control. Bars indicate the mean and standard deviation (SD) of triplicate samples. (**P < 0.005, compared to none treated).

To verify the bone metastatic or tissue-specific effects of TNFα on PTX3 expression, MCF-7, MDA-MB-231, DU-145, and PC-3 cells were utilized. When MDA-MB-231 cells were heated with increasing concentrations of TNFα, expression of PTX3 increased in a dose dependent manner (Figure 2B). By contrast, MCF-7, DU-145, and PC-3 cells showed no significant changes of PTX3 expression in response to TNFα, although DU-145 cells already produce high amounts of PTX3 at basal level (Figure 2A, C, D). Moreover, an analysis of PTX3 protein levels using ELISA showed that PTX3 protein expression was only markedly increased in TNFα-stimulated MDA-MB-231 cells (Figure 2). Levels of PTX3 protein expression in MCF-7, DU-145, and PC-3 cells were not significantly modulated by TNFα (Figure 2). These results suggest that MDA-MB-231 cells produce PTX3 and that this production is enhanced by the proinflammatory cytokine TNFα.

### PTX3 induces breast cancer cell migration, Chemotaxis of macrophages and osteoclast differentiation

**Figure 3:**
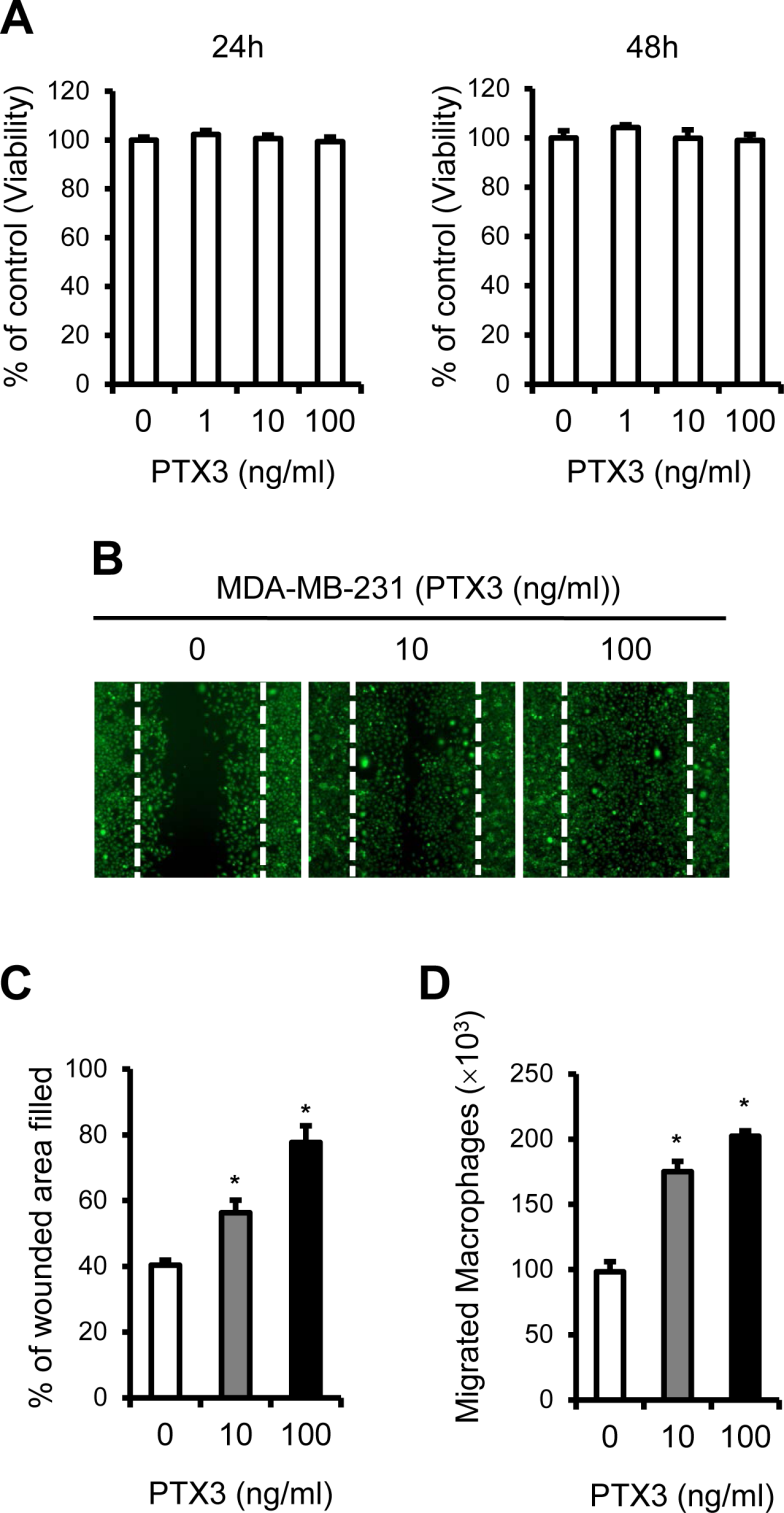
PTX3 enhances breast cancer cell migration and macrophages migration to cancer cells A: Analysis of the effect of PTX3 on cell proliferation in MDA-MB-231 cells. MDA-MB-231 cells were treated with indicated concentrations of PTX3 and proliferative effects of PTX3 on these cells were determined with CCK-8 assay after 24 and 48hours. B: Migration scratch assay to investigate the migration potential of MDA-MB-23 1 cells after treatment with PTX3. Cells were treated with various concentrations of PTX3 (0, 10, 100 ng/ml) and imaged after wound scratching. The dashed lines indicate the lines of the initial wound boundary. C: Wound closure effect was calculated as wound confluence gained in 24 hours. D: Involvement of PTX3 in the migration of macrophages. Recombinant PTX3 proteins (0, 10, and 100 ng/ml) was pretreated for 1 hour to MDA-MB-231 cells at the lower compartments of a transwell system and macrophages (1.2 × 10^6^) were added to the upper chamber of each transwell. Cells were allowed to migrate for 6 hours and the number of macrophages in the lower chamber was counted. Bars indicate the mean and standard deviation (SD) of the numbers of macrophages migrated to lower chamber (*P < 0.05, compared to macrophages with no treatment).

Given that the pro-inflammatory cytokine TNFα up-regulated expression of PTX3, we hypothesized that increased production of PTX3 in breast cancer cells may support cell proliferation and migration. To test this possibility, we examined whether PTX3 regulates breast cancer cell viability and/or proliferation. Cell counting kit (CCK)-8 assays revealed that PTX3 did not affect MDA-MB-23 1 proliferation at 24 and 48 hours (Figure 3A). We next examined whether PTX3 induces migration of MDA-MB-231 breast cancer cells using scratch (wound-healing) assays. As shown in Figures 3B and C, exogenous PTX3 increased the migration capacity of this cell line when compared with untreated cells.

During metastasis, inflammatory cytokines and chemokines enhance infiltration of leukocytes, including macrophages, precursors of OCs [21, 22]. Having shown that MDA-MB-231 cells produce elevated levels of PTX3 compared to MCF-7 cells (Figure 1, 2), we questioned whether PTX3 produced by MDA-MB-231 cells could induce chemotactic migration of macrophages toward breast cancer cells and subsequently enhance osteoclastogenesis. In a transwell assay, MDA-MB-231 cells heated with different concentrations of PTX3 (0, 10, and 100 ng/ml) were incubated in the lower chamber and macrophages were loaded to the upper chamber. Approximately 1.0 × 10^5^ macrophages migrated in controls, but a higher level of cell migration (approximately 2.0 × 10^5^) was observed in the presence of PTX3, suggesting that PTX3 enhanced the migration of macrophages to breast cancer cells (Figure 3D).

**Figure 4:**
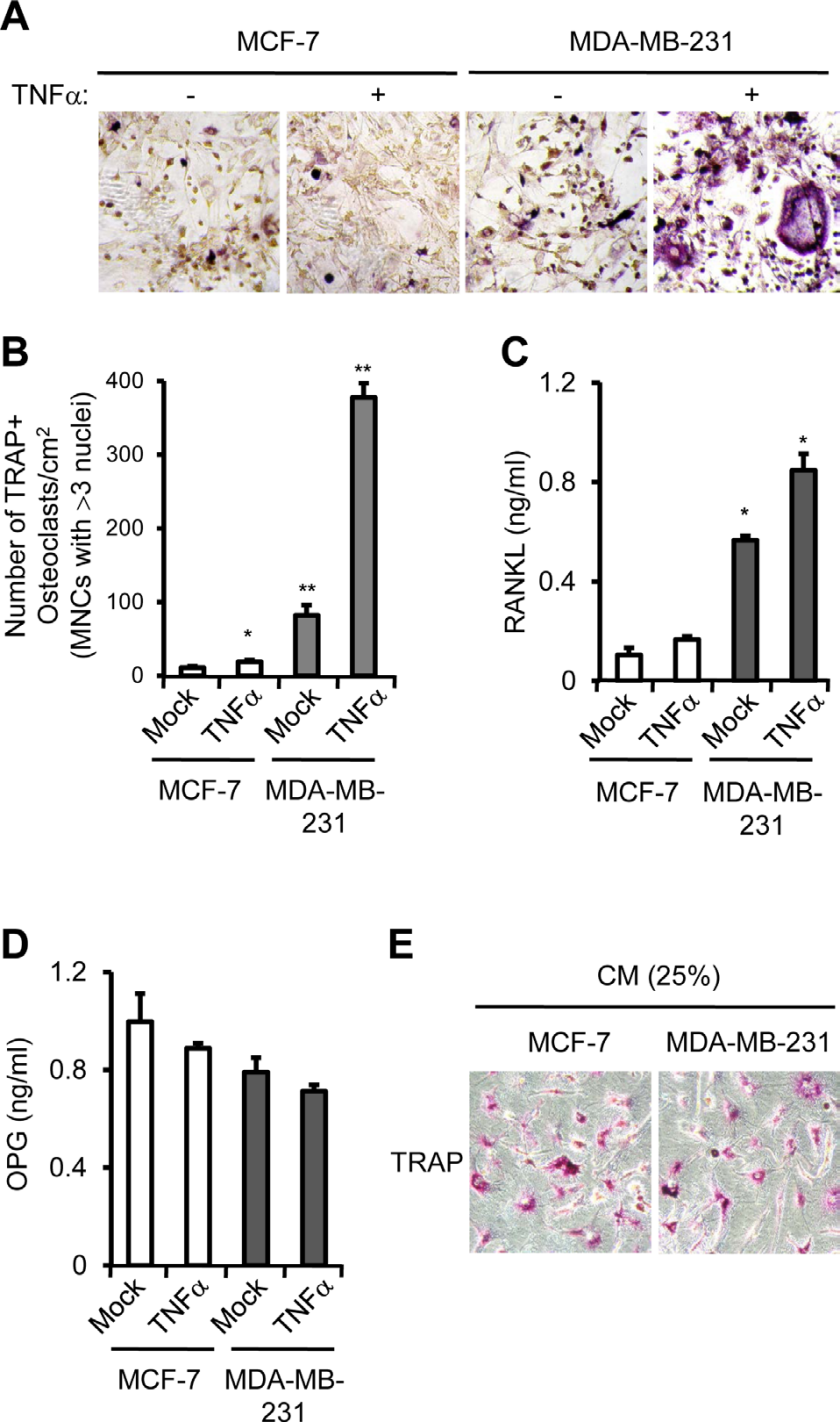
PTX3 derived from breast cancer cell enhances osteoclast differentiation and activation A: Involvement of PTX3 in OC differentiation and activation. Isolated pre-osteoblast cells and bone marrow cells (BMMs) were cultured with Vitamin D3 and PEG2 at the lower compartments of a transwell system. MCF-7 or MDA-MB-231 cells (1.2 × 10^6^) treated with vehicle or TNFα were added to the upper chamber of each transwell. After 7 days, cells were fixed and OCs showing tartrate-resistant acid phosphatase (TRAP) positive (TRAP^+^) multinucleated cells (MNCs) (> 3 nuclei) were identified by TRAP staining under a light microscope. **B:** The numbers of TRAP+MNCs were counted. Bars indicate the mean and standard deviation (SD) of triplicate samples (*P < 0.05; **P < 0.005, compared to MCF-7 cells with none treatment). Soluble RANKT (C) and OPG (D) levels in conditioned media of co-culture from lower compartments of transwell were determined by ETISA. E: Bone marrow derived macrophages were incubated with 25% of conditioned media (CM) of MCF-7 or MDA-MB-231 cells in the presence of M-CSF and RANKT for 5 days and OCs showing TRAP^+^ cells were identified by TRAP staining under a light microscope.

To determine the role of PTX3 in osteolysis, we utilized a co-culture system in which primary murine bone marrow cells were cultured with murine preosteoblasts. This co-culture system represents a simplified version of physiological bone environment, in which OB and OC can interact with each other. We cultured isolated pre-osteoblasts and bone marrow cells in the presence of Vitamin D3 and prostaglandin E_2_ (PEG_2_) at the lower compartments of a transwell system. MCF-7 or MDA-MB-231 cells treated with control solution or TNFα were added to the upper chamber of each transwell to supply PTX3 to lower chamber. The number of tartrate-resistant acid phosphatase (TRAP)-positive multinucleated cells (TRAP+MNCs), i.e., OC formation, resulting from control-treated MDA-MB-231 cells was approximately eight-fold higher than that of MCF-7 (Figure 4A, B). TNF-a treatment further increased OC formation approximately four-fold in MDA-MB-231 cells (*P <* 0.005) compared to the mock (Figure 4B). Because PTX3 did not stimulate OC formation directly (data not shown), we surmised that PTX3 produced by MDA-MB-231 cells may stimulate RANKT production from OBs and subsequently activate OC formation. Thus, we next determined whether the levels of secreted RANKT and OPG proteins from co-culture of OBs and bone marrow-derived macrophages (BMMs) was affected by the presence of MCF-7 or MDA-MB-231 cells. In the presence of vehicle-treated-MCF-7 cells at upper chamber of transwell, approximately 0.1 ng/ml of RANKT was detected in conditioned media using ETISA, and TNFα treatment of these MCF-7 cells did not significantly increased RANKT secretion (Figure 4C). By contrast, RANKT production by the presence of MDA-MB-231 cells at upper chamber of transwell was much higher (~0.56 ng/ml) than that of MCF-7 (~0.1 ng/ml) and was further induced by TNFα treatment (Figure 4C). Expression of osteoprotegerin (OPG), a blocker of RANKT, remained largely unchanged between samples (Figure 4D). These data demonstrate that PTX3 secreted by MDA-MB-231 cells is functionally active in stimulating the chemotactic migration of OC precursor cells (i.e., macrophages) and subsequent OC formation. It should be noted that either TNFα or PTX3 treatment did not influence RANKT expression in breast cancer cells themselves (data not shown), indicating that PTX3 might be involved in OC formation indirectly.

### PTX3 knockdown impaired cancer cell migration, macrophage Chemotaxis to breast cancer cells and subsequent OC formation

**Figure 5:**
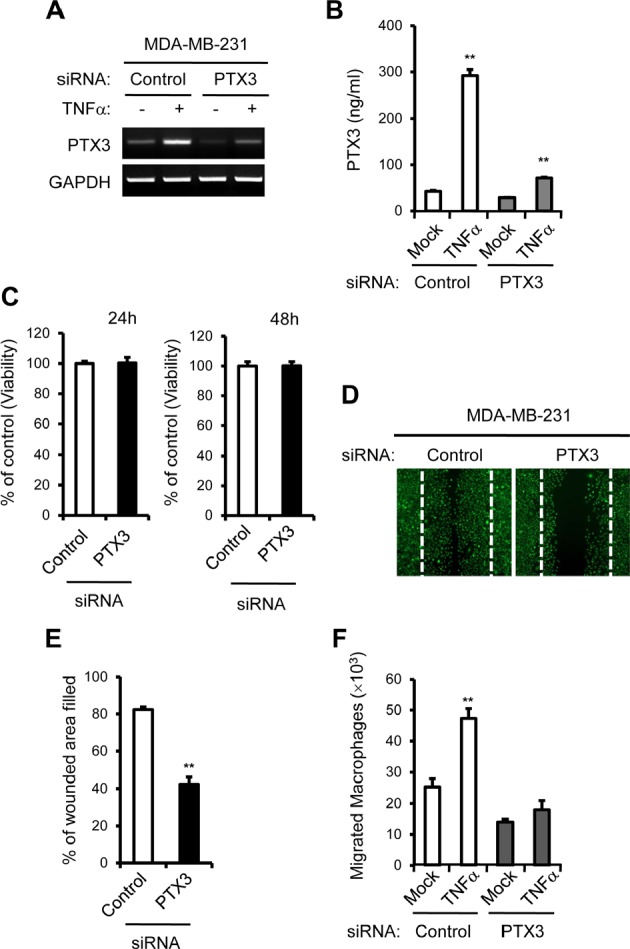
PTX3 knockdown reduces breast cancer cell migration and macrophages migration to cancer cells A: Cells were transfected with either control or PTX3 targeting siRNA followed by treatment with vehicle or TNF-α and the silencing efficacy of PTX3 targeting siRNA was confirmed in MDA-MB-231 cells with RT-PCR. B: PTX3 knockdown was also validated with ETISA methods. C: Analysis of the effect of PTX3 depletion on cell proliferation in MDA-MB-231 cells. D: The effect of PTX3 silencing on MDA-MB-231 cell motility in a wound-healing experiment. MDA-MB-231 cells were transfected with either control or PTX3 targeting siRNA and imaged after wound scratching as in Figure 3. E: Wound closure effect was calculated as wound confluence gained in 24 hours. F: Effect of PTX3 silencing on migration of macrophages. MDA-MB-231 cells at the lower compartments of a transwell system were transfected with either negative control or PTX3 siRNA and then treated with vehicle or TNFα. Macrophages (1.2 × 10^6^) were added to the upper chamber of each transwell and allowed to migrate for six hours and the number of macrophages in the lower chamber was counted. (**P < 0.005, compared to macrophage numbers in non-treated MCF-7 cells).

To confirm the involvement of PTX3 in cell migration, macrophage Chemotaxis, and subsequent OC activation, endogenous PTX3 was knocked down in MDA-MB-231 cells. A combination of three specific small interfering RNAs (siRNAs) targeting PTX3 were introduced to MDA-MB-231 cells, and we analyzed PTX3 mRNA and protein expression after transfection. The expression of PTX3 mRNA was successfully reduced to approximately 30% of the level in MDA-MB-231 cells transfected with control siRNA (Figure 5A). The PTX3 gene silencing was also verified at protein level using ELISA. The secreted PTX3 protein was suppressed by 80% in PTX3 siRNA transfected cells (Figure 5B), demonstrating that PTX3 siRNA efficiently reduced PTX3 expression in MDA-MB-231 cells. We examined the effect of PTX3 deficiency on the proliferation of breast cancer cells. We found that transfection of PTX3 siRNA did not inhibit cell growth in MDA-MB-231 cells (Figure 5C). Next, we performed scratch assays to examine the role of PTX3 in the migration of breast cancer cells. A scratch assay indicated that PTX3-silenced-MDA-MB-231 cells were defective in migration (Figure 5D, E). Furthermore. PTX3 silencing in MDA-MB-231 cells displayed reduced migration of macrophages toward breast cancer cells (Figure 5F).

**Figure 6:**
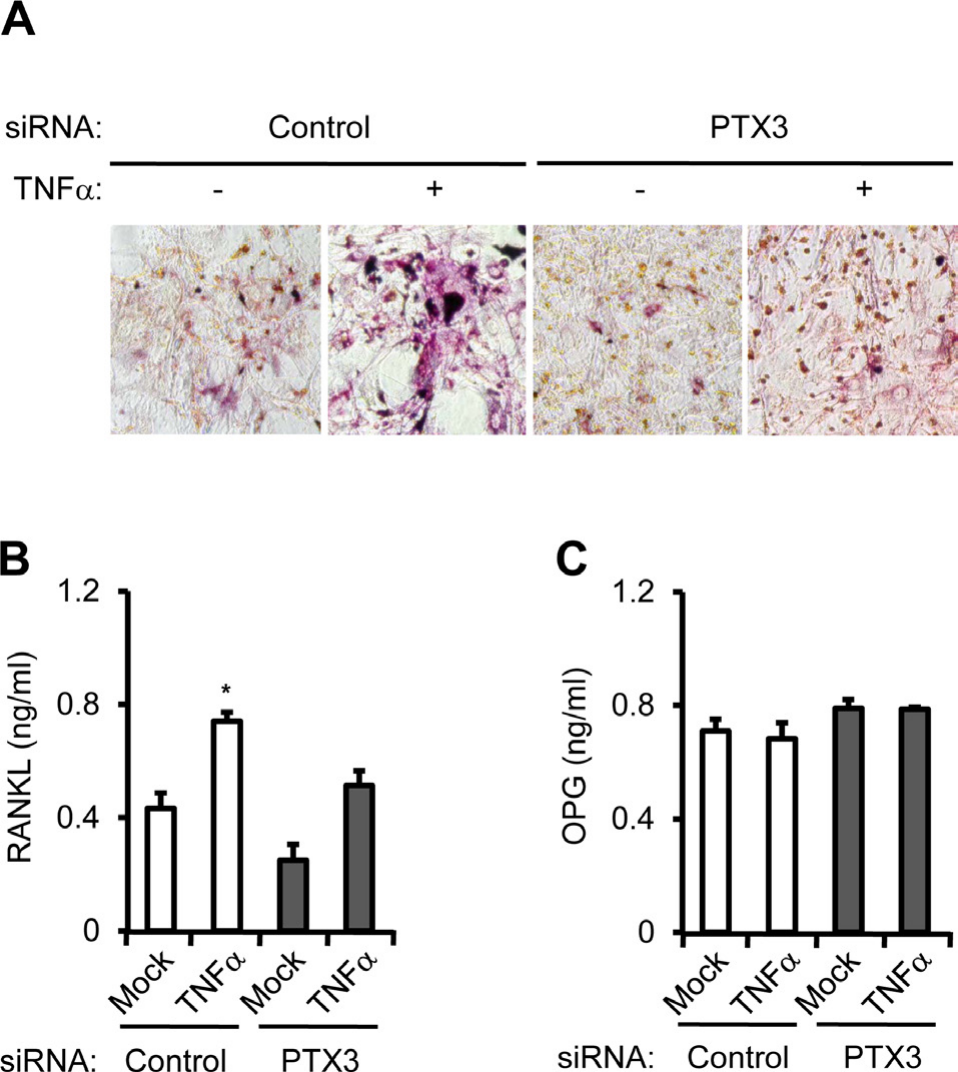
PTX3 knockdown in breast cancer cells reduces OC differentiation and activation A: Involvement of PTX3 in OC differentiation and activation. Isolated pre-osteoblast cells and bone marrow cells were cultured with Vitamin D3 and PEG2 at the lower compartments of a transwell system. MDA-MB-231 cells (1.2 × 10^6^) transfected with negative control or PTX3 siRNA followed by vehicle or TNFα treatment were added to the upper chamber of each transwell. After 7 days, OCs showing TRAP^+^ MNCs (> 3 nuclei) were identified by TRAP staining under a light microscope. The levels of soluble RANKL (B) and OPG (C) proteins from conditioned media of co-culture from lower compartments of transwell were determined by ELISA.

Taken together, when MDA-MB-231 cells were transfected with negative control or PTX3 siRNA and then treated with vehicle or with TNFα, PTX3 silencing in MDA-MB-231 cells led to reduced TRAP+MNC formation, confirming that PTX3 is involved in OC formation (Figure 6A). The levels of secreted RANKL and OPG proteins from co-culture of osteoblasts and BMMs were determined using ELISA. PTX3 silencing in MDA-MB-231 cells transfected with PTX3 siRNA significantly decreased TNF-α-mediated RANKL production in the co-culture system (Figure 6B), but did not modulate OPG production (Figure 6C), supporting the notion that PTX3 may serve as an inflammatory mediator involved in breast cancer-related osteolysis.

## DISCUSSION

Evidence for the close association between the elevated expression of PTX3 and cancer-related inflammation in various malignancies [33–37] suggests its clinical significance. The present study provides the first evidence of the clinical importance of PTX3 as a prognostic factor in bone metastatic breast cancer. A significant up-regulation of PTX3 expression in bone metastasized tumor tissues was detected compared to non-bone metastasized tumor tissues in breast cancer patients (Figure 1A). We observed that increased expression of PTX3 in bone metastatic tumor in human breast cancer patients was accompanied by the direct secretion of extracellular PTX3 by bone metastatic breast cancer cells (Figure 1). In a gene expression study performed to analyze molecular signature of primary breast cancer, levels of PTX3 expression did not differ across low and high-grade breast tumors based on histopathological diagnosis (GEO accession number: GSE6532, GSE9195) (data not shown). This finding was similar to a previous report showing that the level of PTX3 mRNA was not correlated with disease-free survival [43]. However, evaluation of clinical cancer tissue samples of breast cancer patients revealed a weak correlation between PTX3 level and survival (GSE12276) (*p=*0.052). High PTX3 level was significantly correlated with a distant metastasis to bone compared to non-bone tissues of breast cancer (GSE14020) (Figure 1). Thus, PTX3 could be a potentially reliable prognostic biomarker with a tumor-promoting capacity for breast cancer patients with bone metastasis; however, its impact needs further investigation in larger patient groups to assess whether it can evaluate the metastatic potential of breast cancer to bone.

Surprisingly, in prostate cancer cell lines, non-bone metastatic DU-145 cells expressed much higher levels of PTX3 expression compared to bone metastatic PC-3 cells, although PTX3 expression was not responsive to TNFα treatment (Figure 1, 2). In an apparent contradiction of breast cancer behavior, it is known that prostate cancer is osteoblastic (bone-forming) rather than osteolytic (bone-lysing) [44]. This bone-forming phenotype, with excessive OB differentiation, is elicited by prostate cancer cell interactions with OBs and their progenitors, via the production of TGF-β, BMP, IGF, FGF, and WNTs [45]. A previous study reported that PTX3 expression is silenced in prostate cancer cells at a relatively early stage during tumor progression [46]. Since PTX3 acts as a natural antagonist for FGFs [46], the changes in PTX3 level may determine the angiogenic and tumorigenic potential of osteoblastic prostate cancer cells, presumably by inhibiting bone-forming activity exerted by FGFs [46]. It is tempting to postulate that PTX3 may be up-regulated by inflammatory conditions, and may play a unique role in the process of osteolytic cancer cells rather than osteoblastic cancer cells, but further evidence is required to confirm this speculation.

Inflammatory components, such as TNFα, IL-Iβ. and IL-6, are present in the microenvironment of most malignant tissues, including breast tumors [11]. In this study, TNFα treatment led to significant over-expression of PTX3 in bone metastatic breast cancer cells, but not in non-bone metastatic breast cancer cells (Figure 2), reinforcing PTX3 as a modulator of breast cancer-related inflammation. Key features of cancer-related inflammation include NF-κB activation: inflammatory cytokines such as TNFα and IL-Iβ activate NF-κB in both tumor and inflammatory cells, leading to the expression of several mediators [47]. We observed that NF-κB inhibition blocked PTX3 up-regulation induced by TNFα, indicating that NF-κB activation appears to be involved in PTX3 expression when induced by pro-inflammatory cytokine (Figure 1D). Another key feature of cancer-related inflammation is the infiltration of leukocytes, in particular macrophages [48], through enhancing interactions between cancer cells and infiltrating leukocytes [21, 22]. These interactions stimulate to produce several mediators that contribute to the metastasis of breast cancer cells to bone [11, 12]. In line with this, we observed that exogenous PTX3 enhanced the migratory potential of macrophages to breast cancer cells and PTX3 silencing blocked macrophage mobility toward breast cancer cells (Figure 3, 5).

There are multiple biological steps in the distant metastasis of cancer [49]. Before cancer cells reach distant target organs, they grow and invade into the surrounding tissues, and subsequently enter the circulation. During this process, cancer cells obtain enhanced migratory potential by undergoing epithelial-mesenchymal transition (EMT) [33]. Kondo et al. reported that PTX3 protein promoted the migratory potential of pancreatic cancer cells [34]. These data are in agreement with our observations showing that PTX3, increased by TNFα, promoted breast cancer cell migration activity (Figure 3). Further study to define the possible involvement of PTX3 in EMT should be followed.

It is widely accepted that bone resorption mediated by osteolytic breast cancer cells is through the OC-activating cytokines [1, 4], and that the stimulatory effect of cancer on RANKL production by OBs is an important step in the activation of OC [5, 6]. Similarly, PTX3 did not directly induce OC differentiation and activation (Data not shown), whereas PTX3 stimulated RANKL production from OBs leading to enhanced osteoclastogenesis (Figure 4). In this regard, PTX3 may be a critical mediator in the promotion of osteolytic function in bone. It remains unclear how PTX3 increases RANKL expression, because the receptor for PTX3 is not defined. Nevertheless, the stimulatory effect of PTX3 on RANKL production might be a reliable mechanism for osteolysis elicited by metastatic breast cancer cells.

In conclusion, we demonstrated the clinical and biological function of PTX3 in bone metastatic breast cancer, providing evidence that PTX3 has an important role in OC differentiation and activation, leading to the osteolytic properties of breast cancer metastasized to bone. Assessment of PTX3 expression status in breast tumors provides clinically useful prognostic information as a potential biomarker in bone metastatic breast cancer, and indicates that the PTX3 pathway might be a novel therapeutic target for breast cancer metastases to bone.

## METHODS

### Cells and reagents

MCF-7 and MDA-MB-231 human breast cancer cell lines and DU-145 and PC-3 human prostate cancer lines were from American Type Culture Collection (Manassas, VA, USA) and were maintained according to distributors ' instructions. All cells were grown in a 5% CO_2_ atmosphere at 37°C. Pyrrolidine dithiocarbamate (PDTC), SP600125, SB203580, PD98059, and anti-β-actin antibody were purchased from Sigma (St. Louis, MO, USA). Recombinant PTX3, IL-lβ, IL-17, IL-23, IL-34 and TNFα proteins and rabbit anti-human PTX3 antibody for ELISA were from R&D Systems (Minneapolis, MN, USA).

### Total RNA extraction, cDNA synthesis, RT-PCR, and quantitative real-time RT-PCR

Total RNA was extracted from cultured cells using the RNeasy Mini Kit (QIAGEN, Hilden, Germany) according to the manufacturer's protocol. cDNA was synthesized from total RNA using murine Moloney leukemia virus reverse transcriptase (MMTV-RT, GIBCO, Invitrogen, Carlsbad, CA, USA). The cDNAwas amplified using the Takara PCR amplification kit (Takara Biotechnology Shiga, Japan); PCR was carried out in a Perkin Elmer thermal cycler. The sequences of primers used were as follows: PTX3, 5'-CAT CTC CTT GCG ATT CTG TTT TG-3' (sense) and 5'-CCA TTC CGA GTG CTC CTG A-3′ (antisense) and GAPDH (glyceraldehyde 3-phosphate dehydrogenase), 5′-AGC CAC ATC GCT CAG AC A-3′ (sense) and 5′-GCC CAATAC GAC CAA ATC C-3' (antisense). Quantitative real-time RT-PCR (qRT-PCR) was performed using *Power* SYBR Green *1-Step* Kit and an ABI 7000 Real Time PCR System (Applied Biosystems, Carlsbad, CA, USA) according to manufacturer's instructions. The amplification protocol consisted of an initial reverse transcription step at 48°C for 30 minutes, followed by 40 cycles of denaturation at 95°C for 15 seconds and annealing and extension at 60°C for 1 minute. Results are expressed as the ratio of target PCR product relative to GAPDH product. Values were normalized to glyceraldehydes-3-phosphate dehydrogenase (GAPDH) values. Quantitation was performed with RQ manager 1.2 software using the ΔΔCT method (Applied Biosystems. Data are presented as means±standard deviation (SD). All samples were examined in triplicate for each experiment.

### ELISA

PTX3 concentration was measured using a PTX3-specific sandwich ELISA (R&D systems, Minneapolis. MN, USA) in accordance with the manufacturer's protocols. Recombinant human PTX3 serially diluted in culture media was used as a standard. ELISA plates were incubated with mouse anti-human PTX3 antibody (Ab) and then blocked with 3% bovine serum albumin in PBS for 1 hour at room temperature. Conditioned media from cells was added to the plates. After washing with PBS containing 0.05% Tween 20, each well was incubated with biotinylated mouse anti-human PTX3 Ab (0.5 μg/ml) for 2 hours at room temperature. Plates were then washed with PBS containing 0.05% Tween 20 and incubated with streptavidin conjugated to horseradish peroxidase (HRP) for 20 min at room temperature. A color reaction was developed with tetramethylbenzidine solution, and then stopped with 0.1 M H_2_SO_4_. The absorbance at 450 nm was measured using a Bio-Rad microtiter plate reader (Bio-Rad Laboratories, Hercules, CA, USA). RANKL and OPG concentrations in conditioned media were also determined using RANKL- or OPG-specific sandwich ELISA (R&D systems, Minneapolis, MN, USA) according to the manufacturer's protocols. All samples were examined in triplicate for each experiment.

### Gene silencing and transfection

The transfection of small interfering RNA (siRNA) targeting PTX3 was performed as previously described [50]. The combination of four selected sequences of siRNA oligonucleotides, a SMARTpool of siRNA to PTX3 (ON-TARGET plus Human PTX3), and negative control siRNA were purchased from Thermo Scientific Dharmacon (Lafayette, CO, USA). MDA-MB-231 cells were transfected with siRNA using the transfection reagent RNAiMAX (Invitrogen, Carlsbad, CA). Silencing efficiency was evaluated by RT-PCR and ELISA as described above.

### Cell proliferation

Cells were plated in 96 plates for CCK-8 assay (Dojindo Molecular Technologies Inc, Japan). The culture supernatants were removed and replaced with fresh complete culture medium (DMEM) containing various concentrations of PTX3, then culture was maintained for a further 24 or 48 hours at 37°C. At the indicated time, cell growth was assessed by CCK-8 colorimetric assay following the manufacturer's instructions. All analyses were performed in triplicate or more. 10 μl cell counting kit-8 was added to each well and the plates were incubated for an additional 1-4 hours at 37°C. The absorbance at 450 nm of each aliquot was determined using the microplate reader. The effects of PTX3 silencing on cancer cell proliferation were assessed as above.

### Cell migration scratch assays

MDA-MB-231 cells treated with PTX3 or transfected with PTX3 siRNA as above were incubated on 48-well plates. Wounds were generated using a sterile 200-μl pipette tip across each well followed by treatment of recombinant PTX3 (0, 10, and 100 ng/ml) and the cells were incubated at 37°C for an additional 24 hours. For the PTX3 silencing experiment, MDA-MB-231 cells were transfected with negative control or PTX3 siRNA and then the cell layer was scratched. Cells were stained with CellTracker Green (CMFDA) (Invitrogen) according to the manufacturer's instruction and wound closure was assessed using Fluorescence microscopy (LSM700, Carl Zeiss). The percentage of the wounded area filled was measured using Adobe Photoshop 9.0.2 software and compared with baseline measurements. Wound closure was determined as percentage of wound confluence compared to respective negative control (regarded as 100%). Each experiment was performed in triplicates.

### Macrophage isolation and cancer cell migration assay

Isolation of mouse bone marrow cells (BMs) from hind limbs of 6-week-old female RFP transgenic mouse was performed as described previously [51]. Transmigration assays were performed as described previously [52]. MDA-MB-231 (4 × 10^5^) cells were seeded to the lower compartments of a 5-μm pore size transwell system (Costar, Corning, NY, USA) in the presence of various concentrations of PTX3 (0, 10, and 100 ng/ml). Macrophages (2 × 10^6^) were added to the upper chamber of each transwell and allowed to migrate for 6 hours. The number of macrophages in the lower chamber was then counted using fluorescence microscopy (LSM700, Carl Zeiss). For PTX3 silencing experiment, MDA-MB-231 cells in the lower chamber were transfected with either negative control or PTX3 targeting siRNA followed by vehicle or TNF-a treatment. Transwell assays with macrophages in the upper chamber were conducted as above.

### Osteoclast formation in co-culture system

Primary mouse osteoblastic cells were isolated from 1-day-old mouse calvariae after six routine sequential digestions with 0.1% collagenase (GIBCO, Invitrogen, Carlsbad, CA) and 0.2% dispase (Roche, Penzberg, Germany). Following collagenase and dispase digestions, each supernatant was collected and filtered through 70 μm mesh. 3 × 10^5^ cells were cultured on 100 mm plate for 3 days with α-MEM supplemented with 10% FBS and 50 units/ml streptomycin (Life Technologies) and 50 μg/ml penicillin (Life Technologies). For co-culture experiment, MCF-7 or MDA-MB-231 cells treated with TNF-α were loaded on the upper wells. Fresh BMs cells (3 × 10^5^ per well) were cultured with calvarial pre-osteoblasts (2 × 10^4^ per well) on the lower compartments of a 5-μm pore size transwell system (Costar, Corning) in the presence of vitamin D3 (10^−8^M) and PEG2 (10^−6^M) for 5–7 days. The media was changed every 3 days. The cultures were fixed and stained for TRAP (Sigma) to detect OC formation.

### Analyses of PTX3 expression in distant-metastases and survival in primary breast cancer patients using gene expression datasheets

A publicly available breast cancer microarray data sheet (GSE14020) with a known first site of distant relapse was used in the analyses of PTX3 expression. Observed differences with *p*-values <0.05 were considered to be statistically significant (Wilcoxon rank sum test). The survival analysis was performed using breast cancer patient data extracted from the Gene Expression Omnibus (GSE12276). In these patient series, the relapse-free survival was determined from the date of diagnosis to the date of last follow-up. The data were stratified into three groups, samples with high, intermediate and low level of PTX3 expression. Kaplan-Meier plots were drawn by comparing three sample groups using log rank tests. Observed differences with log rank *p*-value <0.05 were considered to be statistically significant.

### Statistical analysis

Comparisons between Kaplan-Meier curves were performed using the log rank test. Wilcoxon rank sum test was performed to compare PTX3 expression in human breast cancer patients. Means ± SDs were calculated for all conditions, and differences between means were analyzed using Student's *t*-test in other comparisons unless otherwise specified. A *p*-value < 0.05 was considered significant.

## Supplementary Figures


