# Supplementary material for: Elevated Pentraxin 3 in bone metastatic breast cancer is correlated with osteolytic function
Source: Oncotarget. 2014 Jan 15;5(2):481–92. doi: 10.18632/oncotarget.1664 (PMC3964223; doi:10.18632/oncotarget.1664)
Supplement: Supplementary file 1 [file oncotarget-05-481-s001.pdf]

Elevated Pentraxin 3 in bone metastatic breast cancer is correlated with osteolytic function – Choi et al

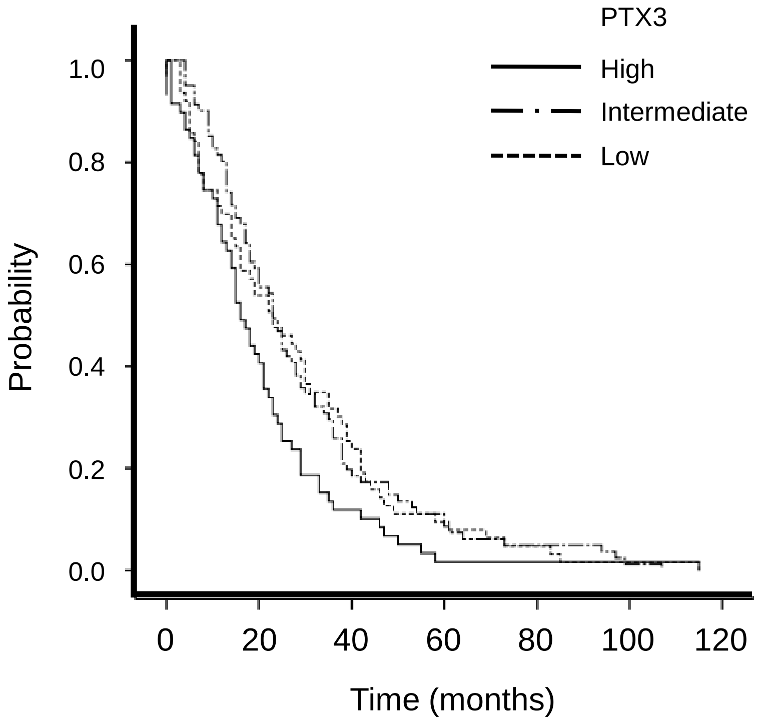

Supplementary Figure 1: Gene expression analysis of PTX3 in clinical breast cancer patients. Kaplan-Meier representation of the probability of cumulative survival in a cohort of 203 breast cancer cases according to PTX3 mRNA expression status as shown as 'High', 'Intermediate', and 'Low'. Tumors are categorized according to PTX3 expression. The data was stratified into three groups, samples with highest, intermediate, and lowest level of expression of the PTX3 gene. Significance of association was determined by log rank test and observed differences with *p*-value <0.05 were considered to be statistically significant.
